# Country data on AMR in Brazil in the context of community-acquired respiratory tract infections: links between antibiotic susceptibility, local and international antibiotic prescribing guidelines, access to medicine and clinical outcome

**DOI:** 10.1093/jac/dkac215

**Published:** 2022-09-06

**Authors:** Didem Torumkuney, Puja Nijhara, Cristiana Ossaille Beltrame, Elisama Queiroz Baisch, Ricardo Macarini Ferreira

**Affiliations:** GlaxoSmithKline, 980 Great West Road, Brentford, Middlesex TW8 9GS, UK; GlaxoSmithKline, 252, Dr Annie Besant Road, Worli 400030, Mumbai, India; GlaxoSmithKline, Estrada dos Bandeirantes, 8464, Jacarepaguá, 22783-110 Rio de Janeiro, Brazil; GlaxoSmithKline, Estrada dos Bandeirantes, 8464, Jacarepaguá, 22783-110 Rio de Janeiro, Brazil; GlaxoSmithKline, Estrada dos Bandeirantes, 8464, Jacarepaguá, 22783-110 Rio de Janeiro, Brazil

## Abstract

**Background:**

Antimicrobial resistance (AMR) is one of the biggest threats to global public health. Selection of resistant bacteria is driven by inappropriate use of antibiotics, amongst other factors. COVID-19 may have exacerbated AMR due to unnecessary antibiotic prescribing. Country-level knowledge is needed to understand options for action.

**Objectives:**

To review the situation with respect to AMR in Brazil and initiatives addressing it. Identifying areas where more information is required will provide a call to action to minimize any further rises in AMR within Brazil and to improve patient outcomes.

**Methods:**

National initiatives to address AMR, antibiotic use and prescribing in Brazil, and availability of susceptibility data, particularly for the key community-acquired respiratory tract infections (CA-RTI) pathogens *Streptococcus pneumoniae* and *Haemophilus influenzae*, were identified. National and international antibiotic prescribing guidelines for CA-RTIs (community-acquired pneumonia, acute otitis media and acute bacterial rhinosinusitis) commonly used locally were also reviewed, along with local antibiotic availability.

**Conclusions:**

In Brazil there have been some initiatives addressing AMR such as the National Action Plan for AMR, established in 2018. Antibiotic consumption in Brazil is high but a ban on over-the-counter sales of antibiotics has led to a decrease in consumption. Local antibiotic susceptibility testing needs to be increased and the Survey of Antibiotic Resistance (SOAR) study in Brazil will provide useful data for pathogens causing CA-RTIs. A more standardized inclusive approach in developing local guidelines, using up-to-date surveillance data of isolates from community-acquired infections in Brazil, could make guideline use more locally relevant for clinicians. This would pave the way for a higher level of appropriate antibiotic prescribing and improved adherence. This would, in turn, potentially limit AMR development and improve clinical outcomes for patients.

## Introduction

Antimicrobial resistance (AMR) is one of the biggest threats to public health throughout the world^1^ as described in the introductory paper of this Supplement.^2^ The WHO states that ‘the world urgently needs to change the way it prescribes and uses antibiotics. Even if new medicines are developed, without behaviour change, antibiotic resistance will remain a major threat’.^3^ The first paper in this Supplement included details about the multiple factors which can drive a rise in AMR along with the global initiatives that are in place to address this threat.^2^ Each country and/or region must also play their part through local initiatives.

To identify how AMR can be addressed in Brazil in the future, it is necessary to review what is happening now. In this paper, we present the current situation in Brazil, determined by using published information (from searching PubMed, Google Scholar and other internet platforms) to ascertain any national initiatives to address AMR, antibiotic use and prescribing in Brazil, and availability of susceptibility data, in particular for the key community-acquired respiratory tract infection (CA-RTI) pathogens *Streptococcus pneumoniae* and *Haemophilus influenzae*. National and international antibiotic prescribing guidelines for CA-RTIs, specifically community-acquired pneumonia (CAP), acute otitis media (AOM) and acute bacterial rhinosinusitis (ABRS), commonly used by healthcare professionals in Brazil were also reviewed, along with how these link to local antibiotic availability. In addition, we aimed to identify areas where more information is required and present a call to action to improve clinical outcomes for patients and to minimize further rises in AMR within Brazil.

## Burden of disease and antibiotic consumption

According to the Global Burden of Disease study in 2015,^4^ lower respiratory tract infections (LRTIs) were the third-highest cause of mortality in Brazil at 47.0 deaths/100 000 people. The study concluded, however, that even though Brazil may experience some socioeconomic difficulties, there was a reduction of the LRTI load, particularly in terms of deaths and disability and also in those under the age of 5 years. The absolute number of deaths in Brazil has increased, but when the mortality rate for CAP is age-standardized, a 25.5% decrease was observed between 1990 and 2015, possibly explained by improved socioeconomics, better healthcare access and antibiotic availability and vaccination.^4^

Between 2000 and 2015 the antibiotic consumption rate in Brazil rose by between 15–20 defined daily doses (DDDs) per 1000 inhabitants per day. Of 76 countries studied, Brazil had the 11th highest rise in consumption, resulting in Brazil ranking 37th out of the 76 countries investigated in terms of DDDs/1000 inhabitants/day.^5^

## Action Plans

Following the formulation by the World Health Assembly in 2015 of a Global Action Plan (GAP) for AMR,^6^ many countries began to develop their own National Action Plan (NAP) and in 2018 the Brazilian Ministry of Health established a NAP for Brazil.^7^ The Brazilian NAP mirrored the strategic objectives of the WHO GAP of 2015, and included: improving awareness and understanding of AMR through effective communication, education, and training; strengthening the knowledge and evidence base on AMR through surveillance and research; reducing the incidence of infection through effective sanitation, hygiene and infection prevention control; optimizing the use of antimicrobials in human, animal, and plant health; and supporting sustainable investment that takes into account of the needs of Brazil, and increasing investment in new medicines, diagnostic tools, vaccines, and other interventions. The current NAP status as reported by the WHO for 2020–21 shows the Brazil NAP at the implementation stage.

The National Policy of Medicines in Brazil is concerned with the public production of medicines, especially those from the Brazilian Essential Medicines List, to ensure adequate access to necessary medicines for the general population. Brazil has a long history of investment in self-sufficient public production of priority medicines.^8^

## Antibiotic prescribing and use

In most countries in Latin America, antibiotics are dispensed only with a prescription in the public sector, but in private pharmacies they can be bought over-the-counter (OTC) without a prescription. It has been suggested that in some situations, such as when there is limited access to a healthcare professional or in resource-limited settings, OTC purchase of antibiotics may be needed, but self-medication risks antibiotic misuse and can result in increasing resistance.^9,10^ Following other Latin American countries, a law was issued in Brazil in 2010, banning OTC sale of antibiotics and enforcing the requirement for a prescription to be provided to, and then retained by, the pharmacy for every antibiotic sale. Before this law was introduced, one study estimated that 46% of antibiotic sales in Brazil were without a prescription.^10^ In São Paulo, Brazil, there was a significant immediate reduction in antibiotic consumption and a reversal of the trend of rising oral antibiotic consumption. Consumption continued to decline the following year such that total antibiotic consumption was less in 2012 than 2008.^9^ Since overuse of antibiotics is recognized as being one of the main drivers of the development of antibiotic resistance, a decline in use is likely to be beneficial in addressing the issue of rising AMR.

## Surveillance

### National surveillance studies

Since 1993, the Pan American Health Organization (PAHO) has been conducting an Epidemiological Surveillance Study known as SIREVA (Sistema de Redes de Vigilancia de los Agentes Responsables de Neumonias y Meningitis Bacterianas), run in Brazil and other Latin American countries and focusing on community-acquired infections.^11^ The study includes *S. pneumoniae* isolates from invasive pneumococcal disease e.g. meningitis and bacteraemia (without a known site of infection). Only a small proportion of the pneumococcal isolates are from pneumonia and so the susceptibility results are not directly relevant to the management of CA-RTIs.^11^

A long-term surveillance programme exists in Brazil, led by the National Health Regulatory Agency (ANVISA), but it compiles data from healthcare-associated infections and a gap remains about overall AMR, especially considering the WHO One Health initiative where not only resistant nosocomial bacteria are monitored but also bacteria from the community.^12^ Between 2016 and 2017, national discussions took place to prepare the Brazilian NAP and an important objective included joining the Global Antimicrobial Resistance and Use Surveillance System (GLASS) and to significantly expand the scope, which resulted in 2017 in Brazil joining GLASS and starting its National Surveillance Program on Antimicrobial Resistance (BR-GLASS).^12^

### Global surveillance studies

#### SOAR

The Survey of Antibiotic Resistance (SOAR) is a multinational antibiotic surveillance study that has been ongoing in an expanding range of countries since 2002. Currently there is an ongoing SOAR study in Brazil which will provide, for the first time, susceptibility data for isolates derived from patients with CA-RTI. The SOAR study aims to collect and make available in published, peer-reviewed papers, local antibiotic susceptibility data, specifically for *S. pneumoniae* and *H. influenzae,* the most commonly isolated pathogen from CA-RTIs.^13^ Key features of the SOAR study are that it focusses on only these pathogens, and that identification and susceptibility testing are performed in an independent centralized laboratory using standardized methodology (CLSI) allowing for comparisons to be made between countries/regions and for the identification of trends over time. SOAR data is analysed based on three different breakpoints: CLSI, EUCAST dose-specific and PK/PD breakpoints.

Clinical breakpoints are cut-off MIC values used to classify microorganisms into the clinical categories susceptible (S), intermediate (I) and resistant (R), in order to assist in the prediction of the clinical success or failure of a specific antibiotic.^14^ Two international organizations define breakpoint values: CLSI and EUCAST. Due to variation in criteria for their definition, there are some differences between CLSI and EUCAST in the clinical breakpoint values for certain bacteria for some antibiotics and this can impact susceptibility interpretation of clinical isolates.^15^ EUCAST breakpoints are dose-specific and use the EMA-approved doses that are included in the Summary of Product Characteristics of an antibiotic. This means that by application of breakpoints for higher doses, the effect of using a raised dose on the clinical efficacy of a particular antibiotic can be predicted. Until 2018, most clinical microbiology laboratories in Brazil applied CLSI breakpoints however, a resolution launched in December 2018 by the Brazilian Ministry of Health implemented Brazilian Committee on Antimicrobial Susceptibility Testing (BrCAST) as the main reference for antimicrobial susceptibility testing in Brazil.^16^ Training has been implemented throughout Brazil to help clinical microbiologists align with the BrCAST guidelines (which are also aligned with the EUCAST guidelines). EUCAST dose-specific breakpoints can be used retrospectively to calculate the susceptibility of previously collected isolates to show the susceptibility levels that would have been achieved at higher doses and so their use can show the effect of increasing the antibiotic dose on the susceptibility of a pathogen, providing additional information so the prescriber can decide if a higher dose would be of benefit. For example, *S. pneumoniae,* the most commonly isolated respiratory pathogen^17,18^ for indications such as CAP, AOM and ABRS, has over time become less susceptible to amoxicillin/clavulanic acid in some countries^19^ since the MICs for some isolates have increased. When treating infections, it is important to be able to eradicate bacterial pathogens with raised MICs to optimize clinical outcome while at the same time minimizing the risk of selecting variants with even higher MICs. This is possible because β-lactams, unlike many other antibiotics, have time-dependent killing properties. Their efficacy depends on the amount of time the drug concentration is present at the site of action. Although increasing the concentration at the infection site over a particular concentration will not have any effect on the efficacy, the use of higher doses and/or more frequent dosing allows for successful eradication of infections caused by pathogens with higher MICs because the time above the MIC is increased.^20^

#### ATLAS and SENTRY

**Figure 1.**
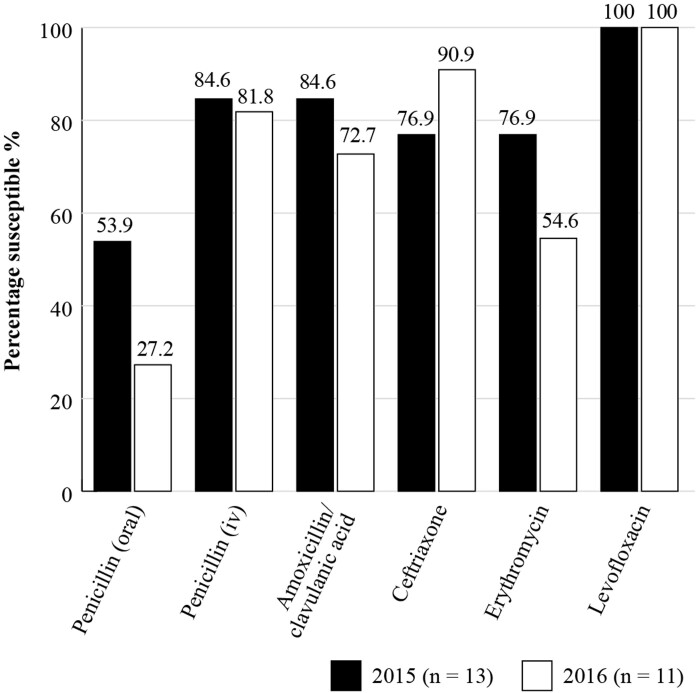
Percentage susceptibility rates based on CLSI breakpoints for antibiotics against *S. pneumoniae* isolates from the ATLAS surveillance programme in Brazil 2015–16. Data access date 21 November 2021.

**Figure 2.**
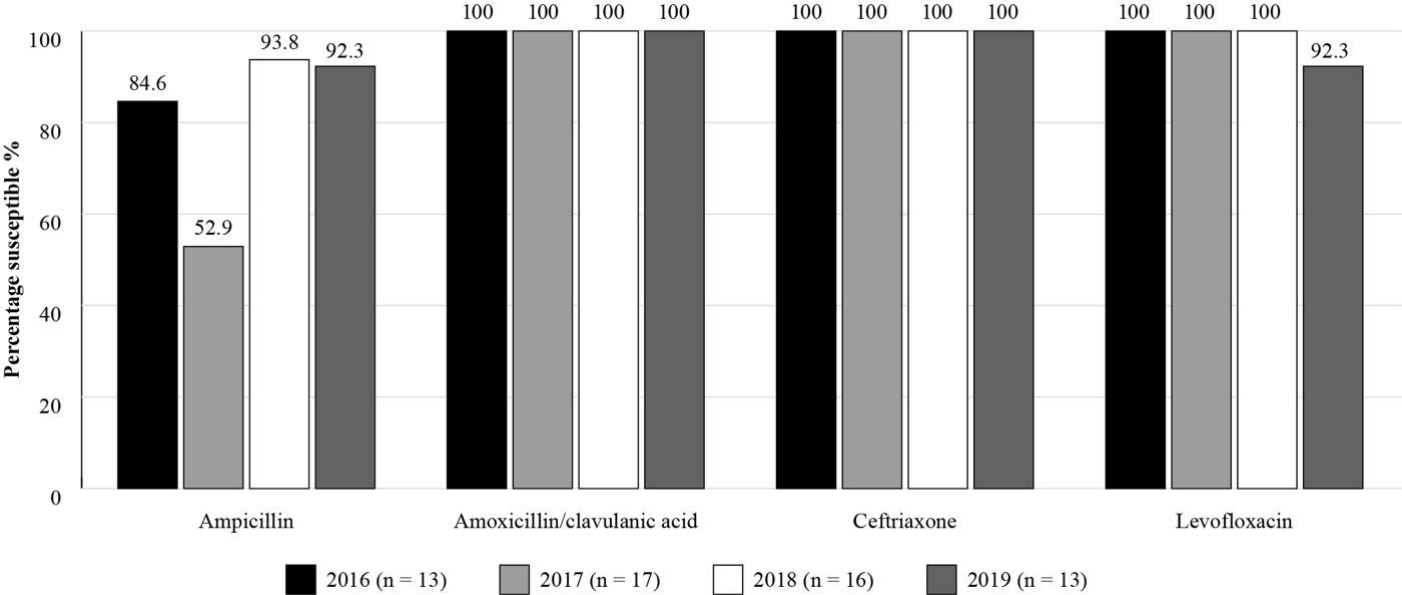
Percentage susceptibility rates based on CLSI breakpoints for antibiotics against *H. influenzae* isolates from the ATLAS surveillance programme in Brazil 2016–19. Data access date 21 November 2021.

The Antimicrobial Testing Leadership and Surveillance (ATLAS) database is a global AMR surveillance programme which is fully accessible, available for general access and covers susceptibilities of a range of bacterial and fungal pathogens to a bank of antimicrobials with reference to the different breakpoints^21^ and susceptibility data are available for Brazil as seen in Figures 1 and 2. Although the number of isolates is very low, in the absence of any data for CA-RTIs in Brazil, the data may be useful.

**Figure 3.**
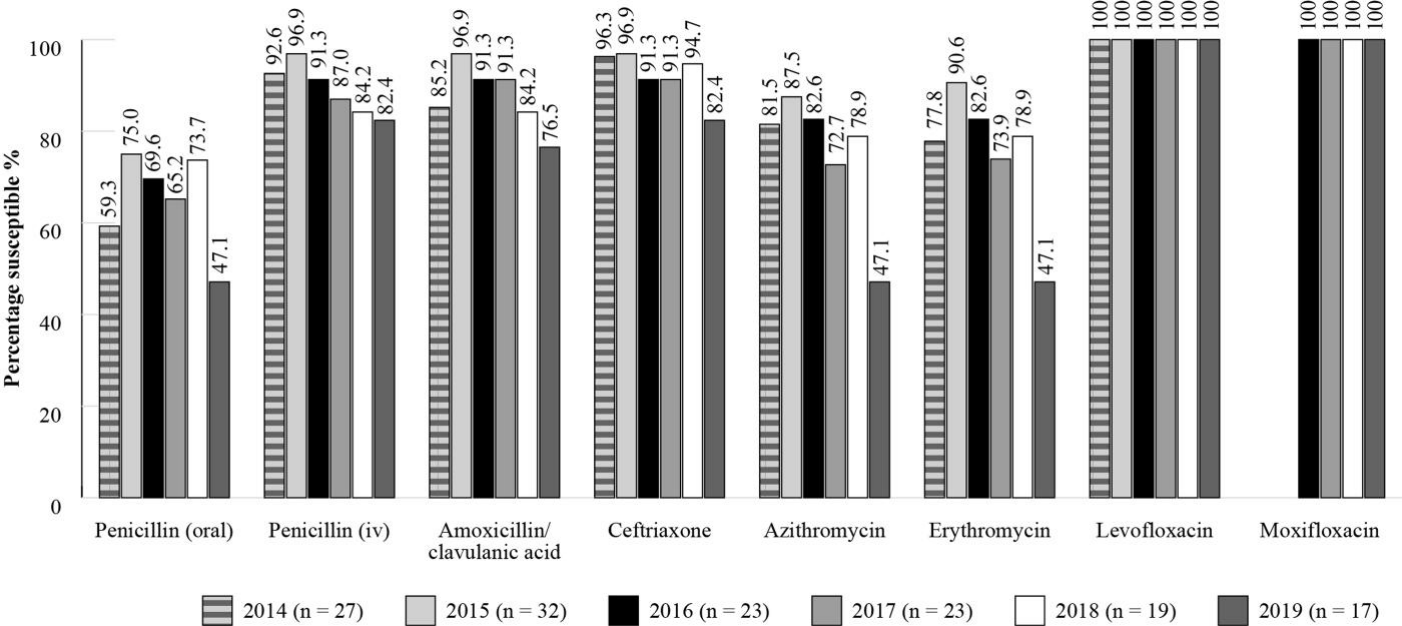
Percentage susceptibility rates based on CLSI breakpoints for antibiotics against *S. pneumoniae* isolates from the SENTRY surveillance programme in Brazil 2014–19. Data access date 21 November 2021.

**Figure 4.**
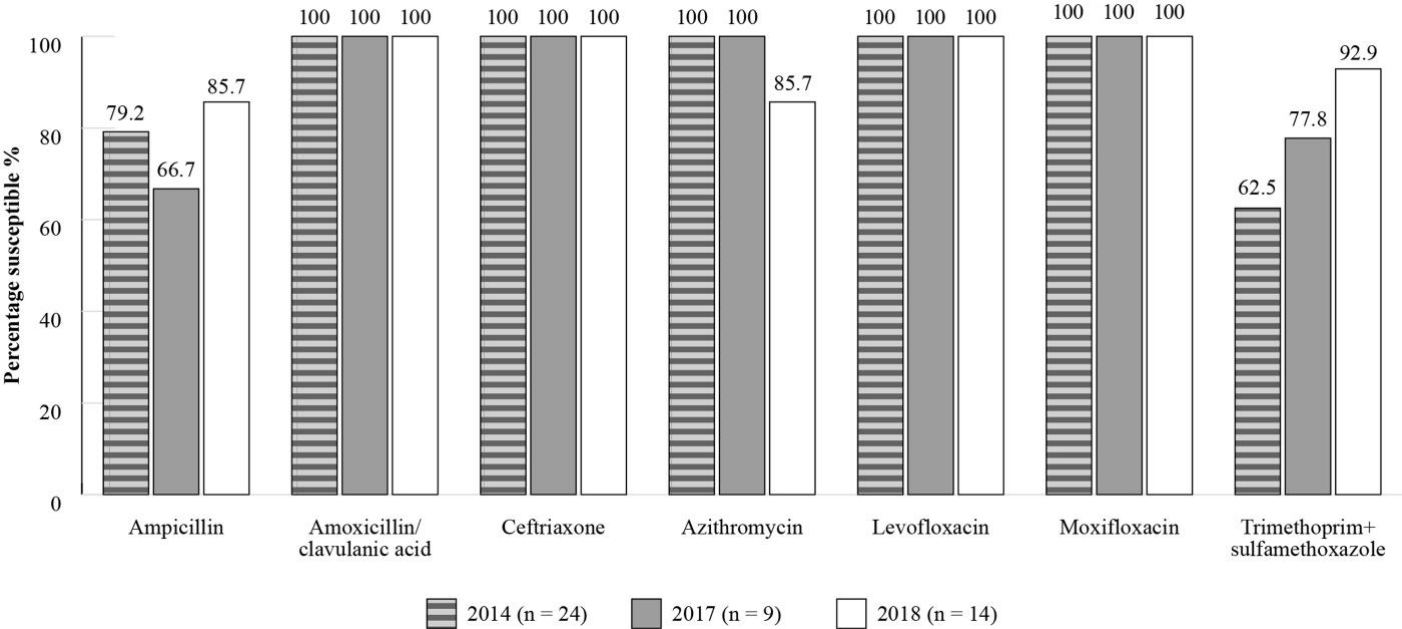
Percentage susceptibility rates based on CLSI breakpoints for antibiotics against *H. influenzae* isolates from the SENTRY surveillance programme in Brazil 2014–18. Data access date 21 November 2021.

The SENTRY Antimicrobial Surveillance Program was designed to monitor the predominant pathogens and AMR for nosocomial and community-acquired infections globally.^22^ Data is available for susceptibility of isolates collected in Brazil although isolate numbers are relatively low (Figures 3 and 4).

Analysis of the results from ATLAS and SENTRY shows that antibiotic resistance in Brazil is increasing with time. Figures 1 and 3 show that prevalence of susceptibility to amoxicillin/clavulanic acid among *S. pneumoniae* isolates was between 72.7%–84. 6% based on ATLAS, and between 76.5%–96.9% based on SENTRY. The data in Figures 1 and 3 also support the view, as described in more detail in the introductory paper to this Supplement,^2^ that resistance to the macrolide antibiotics (erythromycin and azithromycin) is making their use inadvisable. Susceptibility to the fluoroquinolone antibiotics for *S. pneumoniae* has remained high, but great caution concerning their use is now advised due to safety concerns.^23^ This has also been discussed in more detail in the introductory paper.^2^*H. influenzae* remained susceptible to the other antibiotics tested with the exception of ampicillin. ATLAS data is analysed based on both CLSI and EUCAST breakpoints.

#### GLASS

In 2015 the WHO launched GLASS, the first global system to collect national antibiotic resistance data for selected bacterial pathogens that cause common infections. The aim is to monitor the prevalence of antibiotic resistance among major pathogens in clinical settings^24^ to provide the supporting data required to ensure that countries can design cost-effective, evidence-based AMR response strategies. During the first four years, 91 countries/territories have enrolled in GLASS and data for over two million patients from 66 countries were included.^25^ Pathogens currently included in GLASS-AMR are: *Acinetobacter* spp., *Escherichia coli*, *Klebsiella pneumoniae*, *Neisseria gonorrhoeae*, *Salmonella* spp., *Shigella* spp., *Staphylococcus aureus*, and *S. pneumoniae* and a new and important component is the inclusion of antimicrobial consumption (AMC) surveillance at the national level.^26^ GLASS data is analysed based on CLSI and EUCAST breakpoints.

Brazil joined GLASS in 2017, and in 2018, Br-GLASS was initiated. This was a national antimicrobial surveillance programme with the objective of gaining an understanding of the implications of antibiotic resistance in Brazil. 2018 was a pilot year and information was obtained from the clinical microbiology departments of three hospitals. The information included over 200 000 antibiotic susceptibility test results from over 11 000 isolates from over 40 different types of clinical sample. The patients providing the specimens came from over 300 cities, around 56% of patients had community-acquired infections, the remainder were nosocomial. The intention at launch was to cover all five geographical regions in Brazil and to include 95 or more hospitals during the first 5 years.^12^

## Disease Management Guidelines

For management of the common CA-RTIs, CAP, AOM and ABRS in Brazil, clinicians make use of several country-specific local antibiotic prescribing guidelines plus a range of international guidelines (Table 1). Most guidelines suggest a first-line antibiotic or antibiotics along with alternative(s) and then a second-line antibiotic or antibiotics, also with an alternative(s). The first-line antibiotic is the recommended first choice that should be prescribed by the clinician following diagnosis of the infection, supported by the criteria defined by the organization or committee; alternative(s) may be provided for use in particular circumstances. For example, if the first-line antibiotic is a β-lactam then alternative suggestions will be for use in the case of penicillin allergy. The second-line antibiotic is for use if the first-line choice does not achieve the anticipated outcome, and again, alternative(s) may be included for use under specific circumstances.

**Table 1.** Examples of local and international antibiotic prescribing guidelines referred to by physicians in Brazil for the management of community-acquired respiratory tract infections

Local antibiotic prescribing guidelines
Brazilian Guidelines on Rhinosinusitis, 2008^27^
Antimicrobials in Pediatric Clinical Practice. Practical Guide to Management in Outpatient Clinic, Emergency and Nursing 2017^28^
Community-acquired Pneumonia in Children, 2018^29^
Recommendations for the Management of Community-acquired Pneumonia 2018^30^
Diagnostic and Therapeutic Approach for Uncomplicated Community-acquired Pneumonia 2021 no. 6^32^
Complicated Community-acquired Pneumonia 2022 no. 7^31^
International antibiotic prescribing guidelines
IDSA/American Thoracic Society 2007: Infectious Diseases Society of America Guidelines on the Management of community-acquired pneumonia in adults^33^
IDSA 2011 (Endorsed by AAP): The Management of Community-Acquired Pneumonia in Infants and Children Older Than 3 Months of Age: Clinical Practice Guidelines by the Pediatric Infectious Diseases Society and the Infectious Diseases Society of America^34^
IDSA 2012: IDSA Clinical Practice Guideline for Acute Bacterial Rhinosinusitis in Children and Adults^35^
AAP 2013: American Academy of Pediatrics. The Diagnosis and Management of Acute Otitis Media^36^
IDSA 2019: Diagnosis and Treatment of Adults with Community-acquired Pneumonia. An Official Clinical Practice Guideline of the American Thoracic Society and Infectious Diseases Society of America^37^

### International antibiotic prescribing guidelines

For the management of CAP in adults and paediatrics, the international guidelines referred to by clinicians in Brazil include those from the IDSA^34,37^ and from the IDSA/American Thoracic Society (ATS), Consensus Guidelines on the Management of Community-Acquired Pneumonia in Adults (IDSA/ATS).^33^ For example, the first-line recommendation by the IDSA 2019 for treating adults with CAP is amoxicillin and alternatives are doxycycline or a macrolide if local pneumococcal resistance for macrolides is <25%. For adult outpatients with comorbidities and CAP the recommendations are amoxicillin/clavulanic acid, 500 mg/125 mg given three times daily, 875 mg/125 mg or 2000 mg/125 mg both given twice daily or cephalosporins, in combination with a macrolide or doxycycline, or monotherapy with a respiratory fluoroquinolone.^37^

For the management of AOM, the international guidelines referred to in Brazil include those from the American Academy of Pediatrics (AAP)^36^ and for ABRS in adults and paediatrics the international guidelines referred to in Brazil are those from the IDSA.^35^

### National antibiotic prescribing guidelines

For the management of ABRS, AOM and CAP, the commonly used local Brazilian guidelines include the Brazilian Guidelines on Rhinosinusitis, 2008^27^ and Antimicrobials in Pediatric Clinical Practice, Practical Guide to Management in Outpatient Clinic, Emergency and Nursing, 2017.^28^ In CAP, the local guidelines include Community-acquired Pneumonia in Children, 2018^29^ and Recommendations for the Management of Community-acquired Pneumonia 2018.^30^ Most recently, from the Brazilian Society of Pediatrics in 2022, guidelines are provided relating to the management of complicated CAP in children^31^ and the diagnostic and therapeutic approach for uncomplicated CAP, for which the recommendation is amoxicillin, with amoxicillin/clavulanic acid as second-line or if there is a treatment failure.

## Antibiotic availability

Considering amoxicillin/clavulanic acid as an example, in Brazil, several currently available formulations are mentioned as first or second-line recommendations by international RTI management guidelines. This includes the IDSA,^33^ where for outpatients with comorbidities and CAP, the dosing regimens of amoxicillin/clavulanic acid 875 mg/125 mg given twice daily is recommended in addition to 500 mg/125 mg three times daily, for use in combination with a macrolide or doxycycline. In children who are outpatients with presumed pneumonia, amoxicillin/clavulanic acid (amoxicillin component, 90 mg/kg/day twice daily) is recommended by the IDSA as an alternative empirical therapy to first-line amoxicillin treatment for CAP.^34^ In children with AOM, the international guidelines from the American Academy of Pediatrics (AAP)^36^ recommend the amoxicillin/clavulanic acid dose of 90 mg/kg/day twice daily, which is available in Brazil.

Access to antibiotics may be an issue for patients in low- and middle-income countries due to cost and insufficient government expenditure or support in this area. Drug supply chains may also contribute to the problem. Limited access to the most appropriate antibiotic to treat a specific infection may result in raised mortality from treatable bacterial infections, and the use of suboptimal amounts of antibiotic facilitates resistance development and allows resistant strains to persist.^38,39^

Substandard poor-quality or falsified antibiotics promote AMR and the spread of drug-resistant infections.^40^ Since poor-quality antibiotics are unlikely to contain the full dose needed to eliminate all the infecting pathogens this encourages resistance to develop and allows resistant strains to survive and be transmitted.^41^

The quality of medicines, specifically antibiotics, is an important consideration for countries worldwide. The WHO launched a Global Surveillance and Monitoring System (GSMS) for substandard and falsified products.^41^ The GSMS aims to work with WHO member states to improve the quality of reporting of substandard and falsified medical products, and, importantly, to ensure the data collected are analysed and used to influence policy, procedure, and processes to protect public health, at the national, regional and the global level. Use of substandard or falsified antibiotics not only compromises clinical outcome but also risks increased AMR. The most recent summary (2013–17) reported substandard and falsified medicines in 46 member states and antibiotics represent 16.9% of all products reported, second only to malaria drugs (19.6%).

## Conclusions

In an era of rising AMR throughout the world, this paper aims to define areas where action is required to tackle AMR by analysing and understanding the current situation within a country or region. Information is presented for Brazil concerning antibiotic use and prescribing, approach to AMR, availability of local susceptibility data, use of international and/or local management guidelines and how these link to antibiotic availability. To our knowledge this is the first time this information has been reviewed and presented in detail by country.

Antibiotic use in Brazil is extremely high. The burden of infectious diseases is also high in Brazil but studies have shown that the burden of RTIs has reduced in recent years. OTC sales of antibiotics play an important role in increasing the rate of AMR. In 2010 a law was introduced in Brazil prohibiting OTC sales of antibiotics. This resulted in a significant immediate reduction in antibiotic consumption and a reversal of the trend of rising oral antibiotic consumption. In addition, consumption continued to decline the following year so that total antibiotic consumption in Brazil was less in 2012 than 2008.

There is currently a lack of country-wide surveillance studies. The first SOAR study in Brazil is underway and will provide useful data in non-invasive CA-RTIs in the future, but results from global surveillance studies such as ATLAS and SENTRY on the susceptibility of community-acquired respiratory tract isolates reveal some lower levels of susceptibility amongst the common respiratory pathogens for some antibiotic classes, such as the macrolides. The fluoroquinolone antibiotics have so far retained activity, although guidelines and regulatory bodies urge caution, restricting their use to limited situations due to serious safety concerns.

While a range of international and local guidelines are utilized by clinicians in Brazil, a more standardized inclusive approach is needed to develop local country-specific antibiotic prescribing guidelines. These guidelines would be based on up-to-date local surveillance data of isolates from community-acquired infections, which would make them more locally relevant for clinicians, reiterating the Consensus Principles as described in the introductory paper to this Supplement.^2^ This would pave the way for improved adherence and a higher level of appropriate antibiotic prescribing in CA-RTIs which could, in turn, potentially limit AMR development and improve clinical outcomes for patients.
